# The Silencing Effect of microRNA miR-17 on p21 Maintains the Neural Progenitor Pool in the Developing Cerebral Cortex

**DOI:** 10.3389/fneur.2014.00132

**Published:** 2014-07-18

**Authors:** Yase Chen, Shan Bian, Jing Zhang, Haijun Zhang, Beisha Tang, Tao Sun

**Affiliations:** ^1^Department of Neurology, Xiangya Hospital, Central South University, Changsha, China; ^2^Department of Cell and Developmental Biology, Weill Medical College of Cornell University, New York, NY, USA; ^3^School of Life Sciences and Biotechnology, Shanghai Jiao Tong University, Shanghai, China

**Keywords:** miRNAs, miR-17, p21, proliferation, cerebral cortex

## Abstract

Expansion of the neural progenitor pool in the developing cerebral cortex is crucial for controlling brain size, since proliferation defects have been associated with the pathogenesis of microcephaly in humans. Cell cycle regulators play important roles in proliferation of neural progenitors. Here, we show that the cyclin-dependent kinase inhibitor p21 (also called Cdkn1a and Cip1) negatively regulates proliferation of radial glial cells (RGCs) and intermediate progenitors (IPs) in the embryonic mouse cortex. MicroRNA-17 (miR-17) displays reciprocal expressions with p21 in the developing cortex. Opposite to p21, miR-17 promotes expansion of RGCs and IPs, as demonstrated by overexpressing miR-17 precursors and miR-17 sponges that can knock down the endogenous miR-17. Moreover, p21 is a putative target normally silenced by miR-17. Co-expression of miR-17 with p21 is sufficient to rescue the negative regulation of p21 on progenitor proliferation. Our results indicate a mechanism of controlling the neural progenitor pool, which is to suppress p21 by miR-17 in the developing cortex.

## Introduction

Proper proliferation and differentiation of neural stem cells (NSCs) and neural progenitors (NPs) are crucial for the development and function of the mammalian cerebral cortex. Radial glial cells (RGCs) mainly reside in the cortical ventricular zone (VZ), represent early NPs and normally divide symmetrically to generate two RGCs, and asymmetrically to generate one RGC and one intermediate progenitor (IP) or one differentiated neuron (1–7). IPs mostly reside in the subventricular zone (SVZ) and divide symmetrically to generate two differentiated neurons (2, 8–10). The rate and timing of NP proliferation and differentiation are tightly regulated by various cell cycle regulators (11, 12). p21 (also known as Cdkn1a and Cip1) is a cyclin-dependent kinase (CDK) inhibitor that has been shown to mediate the p53-induced G1 checkpoint in cell cycle regulation (13–15). Lack of p21 promotes self-renewal of embryonic and adult NSCs via suppressing the cell cycle exit (16–19). Because proper regulation of NP proliferation is important for controlling brain size, p21 may play a role in determining cortical growth (20). However, how p21 expression levels are precisely controlled in cortical NSCs and NPs remains unclear.

The endogenous small non-coding RNAs, microRNAs (miRNAs), have been shown to play crucial roles in many aspects of cell cycle regulation in the developing nervous system (21–24). miRNAs normally bind to target genes and negatively regulate their expression through imperfect complementary sequence in the 3′-untranslated region (3′UTR) of messenger RNAs (mRNAs) (25, 26). Studies have shown that miRNAs have either reciprocal or overlapping expression patterns with target genes in specific cells and tissues and silence their expression at a posttranscriptional level (27, 28). An important miRNA family is the miR-17–92 cluster, which produces miR-17, 18, 19, and 92, each with conserved seed sequences, and regulates proliferation and survival of various cells (29–33). Our own work and others have shown that miR-17–92 promotes proliferation of cortical NSCs and NPs (34, 35). Since there are six miRNAs produced from the miR-17–92 cluster, and each miRNA usually has multiple targets, the molecular mechanisms of miR-17–92 function in cortical development remain an exciting research topic.

In this study, we show that while p21 negatively regulates NP proliferation by reducing the numbers of both RGCs and IPs in the developing mouse cortex, miRNA miR-17 has an opposite effect on NP development. miR-17 promotes proliferation of RGCs and IPs through suppressing p21 expression. Our studies have identified a mechanism that controls p21 expression levels in NPs *in vivo* by miR-17 during cortical development.

## Materials and Methods

### Animals

CD-1 mice were used for *in utero* electroporation. For staging of embryos, midday of the day of vaginal-plug formation was considered as E0.5; the first 24 h after birth were defined as P0. Animals were maintained at the facility of Weill Cornell Medical College. Animal use was overseen by the Animal Facility and approved by the IACUC at the Weill Cornell Medical College.

### Tissue preparation and immunohistochemistry

Mouse brains were fixed in 4% paraformaldehyde (PFA) in phosphate-buffered saline (PBS) for 1 h at room temperature (RT), incubated in 30% sucrose in PBS overnight at 4°C, embedded in OCT, and stored at −80°C until use. Brains were sectioned (14 μm) using a cryostat. For antigen recovery, sections were incubated in heated (95–100°C) antigen recovery solution (1 mM EDTA, 5 mM Tris, pH 8.0) for 20 min, and cooled down for 20–30 min. Before applying antibodies, sections were blocked in 10% normal goat serum (NGS) in PBS with 0.1% Tween-20 (PBT) for 1 h. Sections were incubated with primary antibodies at 4°C overnight and visualized using goat anti-rabbit IgG-Alexa-Fluor-488, goat anti-chicken IgG-Alexa-Fluor-488, and/or goat anti-mouse or anti-rabbit IgG-Alexa-Fluor-546 (1:300, Molecular Probes) for 1.5 h at RT. Images were captured using a Leica digital camera under a fluorescent microscope (Leica DMI6000B) or a Zeiss LSM510 confocal microscope.

Primary antibodies against the following antigens were used: green fluorescent protein (GFP) (1:1000, Abcam), bromodeoxyuridine (BrdU) (1:50, DSHB), Pax6 (1:500, Covance), and Tbr2 (1:500, Abcam). Cell counting in the mouse cortical tissue was performed on a representative column with the width of 200 pixels in the cortical wall. All sections analyzed were selected from a similar medial point on the anterior–posterior cortical axis.

### Quantitative real-time reverse transcription PCR

Total RNA was isolated from the dorsal cortex of E15.5 mice using RNeasy^®^ Mini kit (Qiagen) according to manufacturer’s instructions, and all samples were treated with DNase to remove genomic DNA. Reverse transcription was performed using Random Hexamer primer (Roche). The quantitative real-time reverse transcription PCR (qRT-PCR) was performed using Power SYBR^®^ Green PCR Master Mix (Life Science) on an Mx4000™ Multiplex Quantitative PCR System (Stratagene) according to manufacturer’s instructions. The RT primers to detect primary transcripts for miR-17 are: F-5′-gaacctcaccttgggactga-3′; R-5′-tgctacaagtgccctcactg-3′. The RT primers to detect p21 are: F-5′-cggtggaactttgacttcgt-3′; R-5′-caatctgcgcttggagtgat-3′.

### *In utero* electroporation

*In utero* electroporation was performed as described (34). Briefly, electroporation was conducted at E13.5 and the brain tissues were harvested 24 h later. Plasmid DNA was prepared using the EndoFree Plasmid Maxi Kit (QIAGEN) according to manufacturer’s instructions, and diluted to 2 μg/μl. For co-expression of p21 and miR-17, the concentrations of p21 and miR-17 are 0.5 and 1.5 μg/μl, respectively, maintaining a total plasmid concentration of 2 μg/μl. DNA solution was injected into the lateral ventricle of the cerebral cortex, and electroporated with five 50-ms pulses at 35 V using an ECM830 electro square porator (BTX).

### Luciferase assays

Neuro2a cells were transfected using Lipofectamine 2000 (Invitrogen) using the manufacturer’s protocol. Plasmids were quantified by UV spectrophotometry and used for transfection in a 2:1 ratio (miRNA: target luciferase constructs); 8:2:1 ratio (sponge:miRNA:target luciferase constructs). pGL4.13 Firefly luciferase (Promega) was used for 3′UTRs of targets. pGL4.73 Renilla luciferase (Promega) was used as a transfection control. Luciferase was measured using the Dual-Luciferase Reporter Assay kit (Promega) using the manufacturer’s protocol and read on a Victor3 1420 multilabel counter (Perkin Elmer). All conditions were run in triplicate, and all experiments were repeated at least once with similar results. Raw data for each condition were normalized for transfection efficiency as the ratio of Firefly luciferase to Renilla luciferase, normalized to the corresponding empty pGL4.13 column to correct for DNA quantification errors, and finally for each luciferase tested, the empty vector control experiment was set to 1 for display.

### Cloning of constructs

Cloning of constructs was done by standard PCR based methods. cDNA from E15.5 C57Bl/6J mice was used. miR-17 isoform precursors were cloned into the pGEM-T vector (Promega) using the following primers: F-5′-cactcgaggtgacagaatttagagctttgg-3′; R-5′-actggacgcagccagtgccg-3′. Mutations of miR-17 in the seed sequence were generated using the QuikChange II Site-Directed Mutagenesis Kit (Agilent Technologies) using the following primers:

F-5′-gtcagaataatgtcgttgtgcttacagtgcaggtagtgatgtgtgcatctactgcagtgagggcacaagtagcattatgctgac-3′;
R-5′-gtcagcataatgctacttgtgccctcactgcagtagatgcacacatcactacctgcactgtaagcacaacgacattattctgac-3′.

Full length cDNA for p21 was cloned using the following primers: F-5′-gactcgaggcaccatgtccaatcctggtgatg-3′; R-5′-cacttcagggttttctcttgcagaag-3′. To knock down p21, the following oligos were used to make short hairpin RNA (shRNA):

m-P21-top1: 5′-agtgtgccgttgtctcttcttcaagagagaagagacaacggcacacttttttt-3′
m-P21-bottom1: 5′-aattaaaaaaagtgtgccgttgtctcttctctcttgaagaagagacaacggcacactggcc-3′
m-P21-top2: 5′-cggtggaactttgacttcgttcaagagacgaagtcaaagttccaccgtttttt-3′
m-P21-bottom2: 5′-aattaaaaaacggtggaactttgacttcgtctcttgaacgaagtcaaagttccaccgggcc-3′
m-P21-top3: 5′-ctttgacttcgtcacggagttcaagagactccgtgacgaagtcaaagtttttt-3′
m-P21-bottom3: 5′-aattaaaaaactttgacttcgtcacggagtctcttgaactccgtgacgaagtcaaagggcc-3′

The 3′UTR fragment for p21 were subcloned into pGL4.13 vector (Promega) using the following primers: F-5′-ataagaatgcggccgccatcttcggccttagccctc-3′; R-5′-gaggactcgggacaatgcag-3′. For luciferase assays, the miR-17 precursor and its mutation were subcloned into the pCDNA3.1 vector.

For cloning miR-17 sponge, the following primers were used: 5′-gtaggaatcttcgaaagctatacaccgctcgagactagtctacctgcactgccgcactttggttatcctacctgcactgccgcactttggttatcctacctgcactgccgcactttgtctagagcttacgttagaatcgcattcg-3′

For cloning miR-17 sponge mutations, the following primers were used: 5′-gtaggaatcttcgaaagctatacaccgctcgagactagtctacctgcactgccgccgcttggttatcctacctgcactgccgccgcttggttatcctacctgcactgccgccgcttgtctagagcttacgttagaatcgcattcg-3′

They were then subcloned into the pCAGIG vector for electroporation, and pCDNA3.1 vector for luciferase assays.

### Western blot analysis

Expression levels of p21 were analyzed by the western blot analysis. Protein extracts were harvested by lysing N2a cells transfected with combinations of p21 and shRNA for p21 with RIPA lysis buffer (150 mM NaCl, 1 mM Na4P2O7, 1 mM NaF, 1 mM EDTA, 1 mM PMSF, 2 mM Na3VO4, 1% NP-40, 50 mM Tris, pH 7.5) with complete™ EDTA-free protease inhibitor mixture (Roche Diagnostics, Indianapolis, IN, USA). The protein samples were boiled in SDS sample buffer for 10 min before loading onto 10% Tris-Glycine gels as 10 μg for each lane and transferred onto PVDF membrane (Pall Corporation, Pensacola, FL, USA). For immunoblotting, membranes were blocked with 5% (w/v) non-fat milk powder in 0.05% TBST [50 mM Tris-Cl, pH 7.5, 150 mM NaCl, with 0.05% (v/v) Tween-20] and incubated at 4°C overnight with the following primary antibodies, which were diluted in 0.05% TBST with 5% non-fat milk: p21 and actin. After washing with TBST, membranes were incubated with specific HRP-conjugated secondary antibodies for 1 h at RT followed with extended washes with TBST. Immunoblot reactions were visualized using chemiluminescent substrate (Pierce, Rockford, IL, USA) on Kodak BioMax light films (Kodak, Rochester, NY, USA).

## Results

### p21 negatively regulates expansion of RGCs and IPs in embryonic cortices

**Figure 1:**
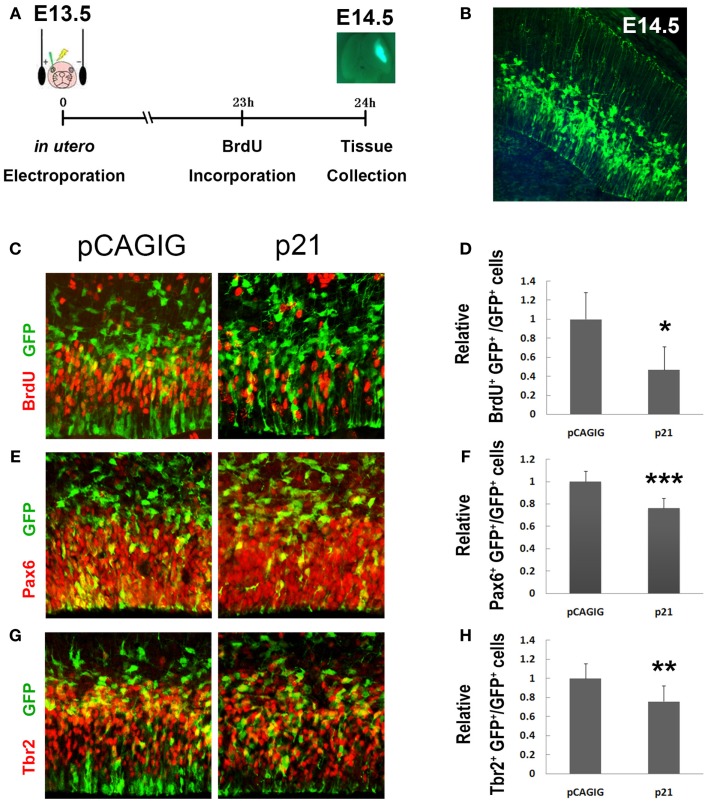
**p21 negatively regulates proliferation of cortical neural progenitors**. **(A)** The electroporation was conducted at E13.5 and the brain tissues were harvested 24 h later. A single intraperitoneal injection of BrdU was performed 1 h before tissue collection. **(B)** The cells located in the ventricular zone (VZ) of mouse cortices were electroporated with exogenous plasmids carrying green fluorescent protein (GFP) at E13.5. The GFP cells can be visualized under microscope at E14.5. **(C,D)** Overexpressing p21 in E13.5 mouse cortices (*n* = 4), analyzed at E14.5, decreased the number of proliferating cells co-labeled with GFP and BrdU compared with the empty vector pCAGIG (*n* = 4). **(E,F)** Overexpressing p21 (*n* = 6) decreased the number of radial glial cells (RGCs) co-labeled with GFP and Pax6 compared with the empty vector pCAGIG (*n* = 4). **(G,H)** Overexpressing p21 (*n* = 5) decreased the number of intermediate progenitors (IPs) co-labeled with GFP and Tbr2 compared with the empty vector pCAGIG (*n* = 4). Data are presented as mean ± SEM; *n* ≥ 3 in all constructs; *p* values in relation to the control (*p < 0.05, **p < 0.01, ***p < 0.001).

Previous studies have shown that p21 usually inhibits self-renewal of NSCs (16–19). To test whether p21 specifically affects proliferation of RGCs and IPs, we examined p21 effects by altering its expression levels in mouse embryonic cortices utilizing *in utero* electroporation (Figure 1A). To overexpress p21, mouse *p21* full length cDNA was cloned into a pCAGIG vector with a GFP reporter gene. The electroporation was conducted at embryonic day 13.5 (E13.5) and brain tissues were harvested 24 h later, with a single pulse of BrdU 1 h before tissue collection (Figure 1A). The electroporated cells in E14.5 cortices were detected by the GFP activity in green (Figure 1B).

A significantly decreased percentage of BrdU^+^/GFP^+^ cells versus total GFP^+^ cells in cortices was detected, suggesting a reduced proliferating NP population when p21 is overexpressed (Figures 1C,D). We then examined the impact of overexpressed p21 on producing RGCs and IPs, by labeling with antibodies against Pax6 – a marker for RGCs, and Tbr2 – a marker for IPs, respectively. There was a significant decrease in percentages of Pax6^+^/GFP^+^ and Tbr2^+^/GFP^+^ cells versus total GFP^+^ cells, indicating a suppression of maintenance of RGCs and IPs by p21 overexpression (Figures 1E–H).

**Figure 2:**
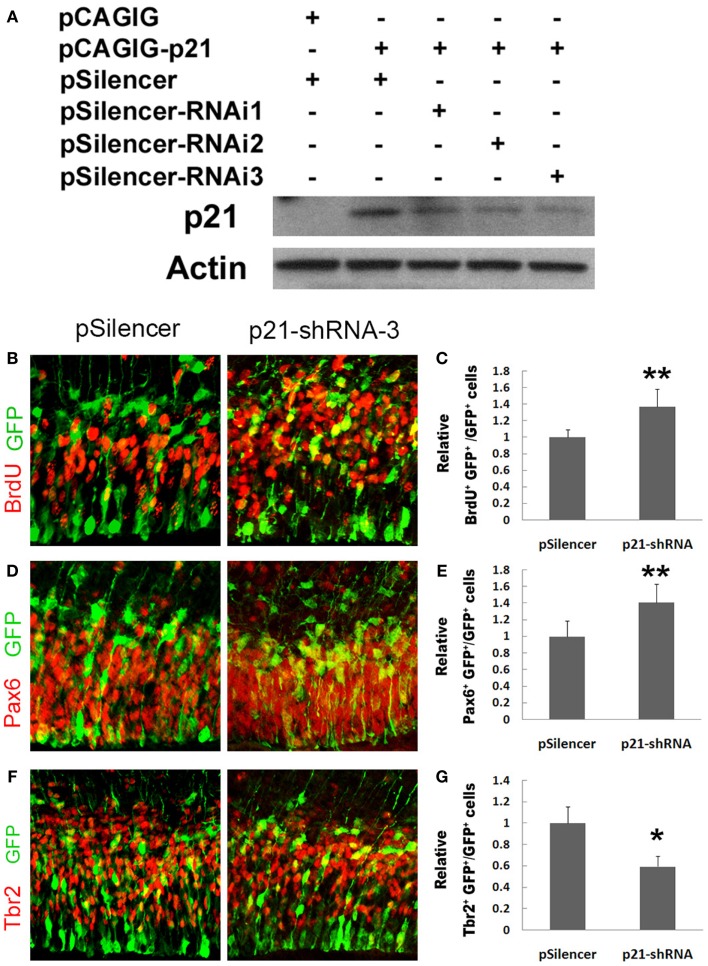
**Knockdown of p21 increases proliferation of cortical neural progenitors**. **(A)** Western blotting analyses of p21 short hairpin RNA to knock down endogenous p21 expression levels using RNA interference (RNAi). **(B,C)** Blocking the p21 expression (*n* = 4) using a p21 short hairpin RNA-3 (p21–shRNA-3) in E13.5 mouse cortices, analyzed at E14.5, increased the number of proliferating cells co-labeled with GFP and BrdU compared with the empty vector pSilencer (*n* = 3). **(D,E)** Blocking the p21 expression (*n* = 5) using the p21–shRNA increased the number of RGCs co-labeled with GFP and Pax6. **(F,G)** Blocking the p21 expression (*n* = 6) using the p21–shRNA decreased the number of IPs co-labeled with GFP and Tbr2. Data are presented as mean ± SEM; *n* ≥ 3 in all constructs; *p* values in relation to the control (*p < 0.05, **p < 0.01).

We next used a short hairpin RNA to knock down endogenous p21 expression (Figure 2A). When the endogenous p21 expression was knocked down using p21 short hairpin RNA-3 (p21–shRNA-3), we found a significant increase in the percentage of BrdU^+^/GFP^+^ cells (Figures 2B,C). The percentage of Pax6^+^/GFP^+^ cells was also greatly increased, suggesting expanded RGC populations (Figures 2D,E). Interestingly, the percentage of Tbr2^+^/GFP^+^ cells was decreased, suggesting that knockdown of p21 affects transition of RGCs to IPs (Figures 2F,G). Our results indicate that p21 negatively regulates proliferation of cortical NPs, and affects expansion and transition of RGCs and IPs.

### p21 is a putative target of miR-17

**Figure 3:**
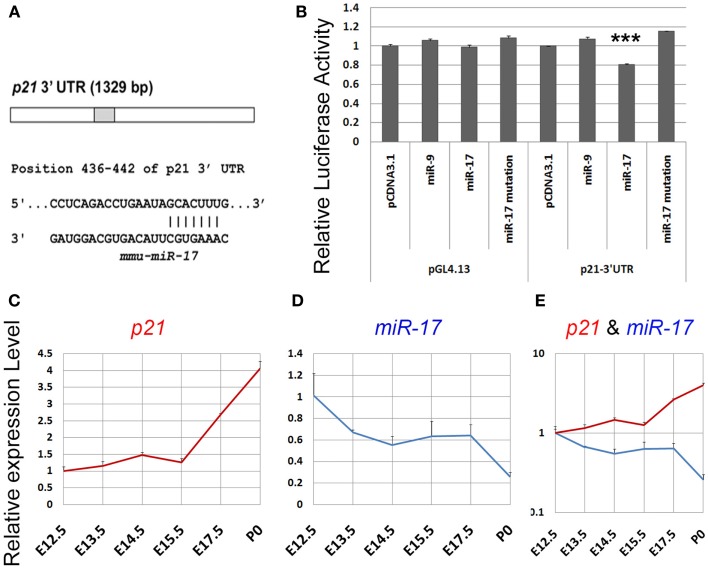
**p21 is a putative target of miR-17**. **(A)** A predicted targeting site of miR-17 on the 3′UTR of *p21*. **(B)** Luciferase activities of the *p21* 3′UTR construct were greatly reduced by miR-17, but not by miR-17 mutation or a control microRNA miR-9. miR-17 had no effect on pGL4.13 construct in which the *p21* 3′UTR had been removed. **(C)** The expression level of p21 in the mouse cortex continuously increased from E12.5 to P0. **(D)** The expression level of miR-17 in the mouse cortex continuously decreased from E12.5 to P0. **(E)** A combined view of the expression levels of p21 and miR-17 at different developing stages. Data are presented as mean ± SEM; *n* = 3; ***p < 0.001.

To maintain a proper population of NPs in early developing cortices, it appears that p21 expression should be precisely modulated, especially repressed. We postulated that miRNA-mediated gene silencing regulation might be one of the mechanisms controlling p21 expression. Based on miRNA prediction algorithm Targetscan (http://www.targetscan.org), we searched the 3′UTR of *p21* and found that it contains one binding site for miR-17 (Figure 3A). To prove the targeting effect of miR-17 on p21, we designed a luciferase assay in which the 3′UTR sequence of *p21* was cloned into a luciferase vector and co-transfected with miR-17. While relative luciferase activities in constructs containing the 3′UTR of *p21* were not affected by a control miRNA miR-9, they were significantly reduced by miR-17 (Figure 3B). However, when a mutated miR-17 containing mutations in the seed sequence, which is responsible for binding to *p21* 3′UTR, was used, the targeting effect of miR-17 was abolished (Figure 3B). These results indicate that p21 is a specific target of miR-17.

We next examined whether the expression level of p21 is correlated with miR-17 expression in developing cortices by performing qRT-PCR. Total RNAs were extracted from E13.5, E14.5, E15.5, E17.5, and postnatal day 0 (P0) cortices. While the expression level of miR-17 was decreased from E12.5 to P0, p21 expression was continuously increased, suggesting a reciprocal expression between miR-17 and its target p21 during cortical development (Figures 3C–E). These results suggest that miR-17 silencing regulation of p21 likely controls NP population in embryonic cortices.

### miR-17 positively regulates proliferation of cortical neural progenitors

**Figure 4:**
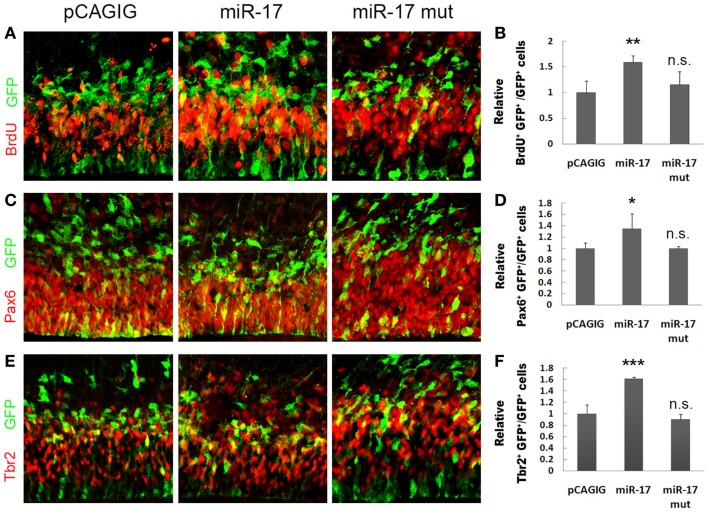
**miR-17 positively regulates proliferation of cortical neural progenitors**. **(A,B)** Expression of miR-17 in E13.5 mouse cortices (*n* = 3), analyzed at E14.5, increased the number of proliferating cells co-labeled with GFP and BrdU. A mutated miR-17 (miR-17 mut) (*n* = 3) had no effect on cell proliferation. **(C,D)** Expression of miR-17 in E13.5 mouse cortices (*n* = 4) increased the number of RGCs co-labeled with GFP and Pax6. A mutated miR-17 (*n* = 3) had no effect on the number of RGCs. **(E,F)** Expression of miR-17 in E13.5 mouse cortices (*n* = 4) increased the number of IPs co-labeled with GFP and Tbr2. A mutated miR-17 (*n* = 3) had no effect on the number of IPs. Data are presented as mean ± SEM; *n* ≥ 3 in all constructs; *p* values in relation to the control (*p < 0.05, **p < 0.01, ***p < 0.001), n.s., not significant.

We next investigated the effect of miR-17 on NP development *in vivo*. In E14.5 cortices, electroporated with the miR-17 precursor to overexpress miR-17 at E13.5, there was a significant increase in the percentage of BrdU^+^/GFP^+^ cells, which suggests an up-regulation of proliferation of cortical NPs (Figures 4A,B). Moreover, the percentages of Pax6^+^/GFP^+^ and Tbr2^+^/GFP^+^ cells were also increased, indicating an expansion of RGCs and IPs by miR-17 overexpression (Figures 4C–F). On the other hand, mutations of miR-17 in the seed sequence (miR-17 mut) had no effect on proliferation of RGCs and IPs, indicating a specific effect of miR-17 on NP development (Figures 4C–F). Our results suggest that miR-17 promotes proliferation of cortical NPs.

### Blocking miR-17 using its sponge causes reduced proliferation of cortical neural progenitors

**Figure 5:**
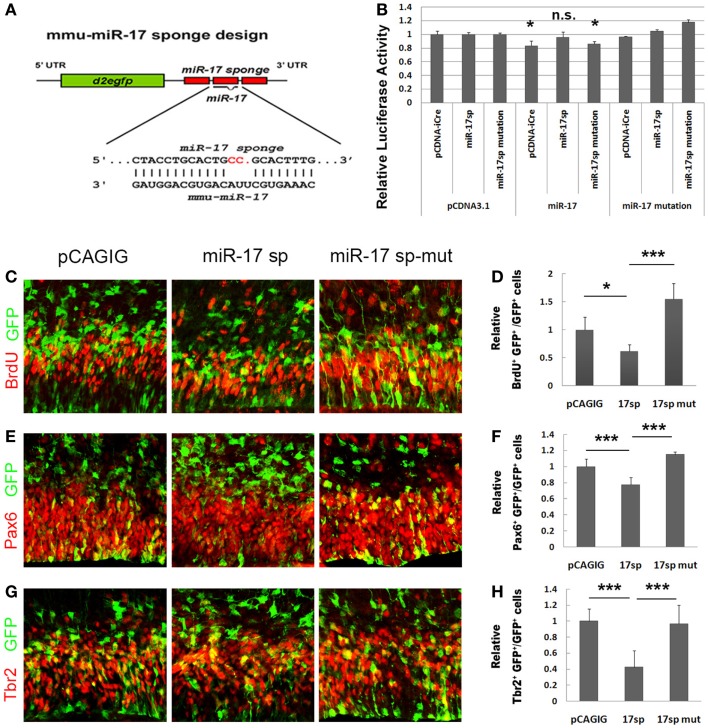
**Blocking miR-17 using its sponge causes reduced proliferation of cortical neural progenitors**. **(A)** The design of the miR-17 sponge. **(B)** The reduction of the relative luciferase activity caused by miR-17 was significantly rescued by the miR-17 sponge, but not by miR-17 sponge mutation. The luciferase activity values were normalized by the average luciferase activity value of the empty vector pcDNA to get a ratio. In all experiments, the pcDNAs luciferase activity value was normalized as 1, and the values of other constructs were the ratios of their luciferase activity values to their pcDNA controls. **(C,D)** Expression of miR-17 sponge (miR-17 sp) in E13.5 mouse cortices (*n* = 4), analyzed at E14.5, decreased the number of proliferating cells co-labeled with GFP and BrdU. A mutated miR-17 sponge (miR-17 sp-mut) (*n* = 3) failed to do so. **(E,F)** Expression of miR-17 sponge in E13.5 mouse cortices (*n* = 6) decreased the number of RGCs co-labeled with GFP and Pax6. A mutated miR-17 sponge (*n* = 3) failed to do so. **(G,H)** Expression of miR-17 sponge in E13.5 mouse cortices (*n* = 4) decreased the number of IPs co-labeled with GFP and Tbr2. A mutated miR-17 sponge (*n* = 4) failed to do so. Data are presented as mean ± SEM; *n* ≥ 3 in all constructs; *p* values in relation to the control (*p < 0.05, ***p < 0.001).

To further test miR-17 functions in expansion of cortical NPs, we applied a loss-of-function approach by expressing miR-17 sponges (36). A miRNA sponge is a RNA transcript that contains multiple binding sequences complementary to a mature miRNA. It can bind to endogenous miRNAs and block their silencing activity (37, 38). We designed the miR-17 sponge (miR-17 sp), which consists of three narrowly spaced, bulged binding sites for miR-17 (Figure 5A). To verify the blocking effect of miR-17 sponge on the miR-17 silencing activity, we performed the luciferase assay. The miR-17 sponge was cloned in the 3′UTR of a coding gene *iCre*, and co-transfected with miR-17 and the construct containing the *luciferase* gene followed by the 3′UTR sequence of *p21*. The reduction of the relative luciferase activity, which is normally caused by the silencing effect of miR-17, was significantly rescued by the miR-17 sponge (Figure 5B). However, a mutated miR-17 sponge at the seed sequence showed no such rescue effect. These data indicate that the miR-17 sponge is able to block the silencing function of miR-17 to its target gene p21.

To further test the effect of miR-17 sponge on expansion of cortical NPs *in vivo*, the miR-17 sponge was electroporated into the cortices of E13.5 mice, and analyzed at E14.5. The percentages of BrdU^+^/GFP^+^, Pax6^+^/GFP^+^, and Tbr2^+^/GFP^+^ cells versus total GFP^+^ cells were all decreased significantly when the miR-17 sponge was expressed (Figures 5C–H). The mutated miR-17 sponge (miR-17 sp-mut), however, did not show effects to the RGCs and IPs, compared to the empty vector and miR-17 sponge (Figures 5C–H). These results indicate that the miR-17 sponge, which can block the endogenous miR-17 silencing activity, suppresses proliferation of cortical NPs.

### miR-17 rescues the negative effect of p21 on proliferation of neural progenitors

**Figure 6:**
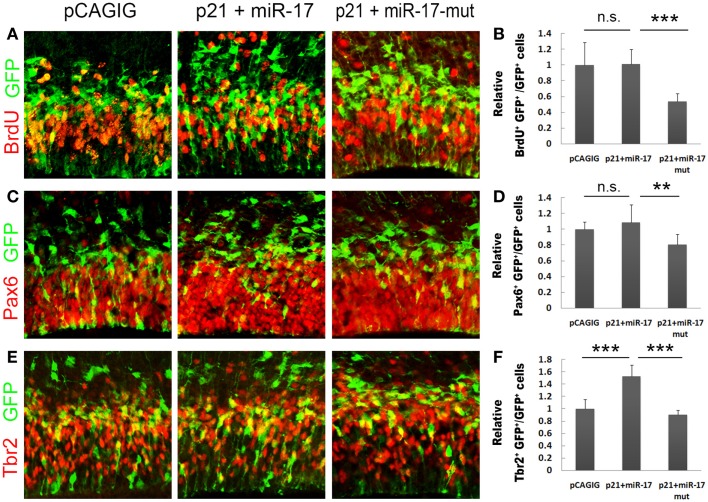
**miR-17 rescues the negative effect of p21 on proliferation of neural progenitors**. **(A,B)** Co-expression of miR-17 with p21 in E13.5 mouse cortices (*n* = 6), analyzed at E14.5, significantly rescued the reduced number of proliferating cells caused by p21 alone, while co-expression of a mutated miR-17 with p21 (*n* = 3) failed to do so. **(C,D)** Co-expression of miR-17 with p21 (*n* = 5) significantly rescued the reduced number of RGCs caused by p21 alone, while co-expression of a mutated miR-17 (miR-17-mut) with p21 (*n* = 3) failed to do so. **(E,F)** Co-expression of miR-17 with p21 (*n* = 5) significantly rescued the reduced number of IPs caused by p21 alone, while co-expression of a mutated miR-17 with p21 (*n* = 3) failed to do so. Data are presented as mean ± SEM; *n* ≥ 3 in all constructs; *p* values in relation to the control (**p < 0.01, ***p < 0.001), n.s., not significant.

To directly test the silencing activity of miR-17 on p21 *in vivo*, we speculated that co-expression of miR-17 with p21 should be able to rescue the negative effect of p21 on proliferation of NPs. The *p21* full length cDNA with the 3′UTR was cloned into the *pCAGIG* vector, co-electroporated with miR-17 into E13.5 cortices, and analyzed at E14.5. Compared to p21 alone, co-expression of miR-17 and p21 significantly rescued NP proliferation, as demonstrated by increased percentages of BrdU^+^/GFP^+^, Pax6^+^/GFP^+^, and Tbr2^+^/GFP^+^ cells, which were compatible to those electroporated with the control vector (Figures 6A–F). However, co-expression of p21 and the mutated miR-17 failed to rescue NP proliferation (Figures 6A–F). These results are consistent with those of the luciferase assay, and prove that miR-17 blocks the negative effect of p21 on proliferation of NPs *in vivo*, especially RGCs and IPs in developing cortices.

## Discussions

Accumulating evidence has shown that protein-coding genes and miRNAs play indispensable roles in mammalian neural development. In our studies, we have identified p21 as a negative regulator of RGC and IP expansion in the mouse embryonic cortex. Utilizing *in utero* electroporation and miRNA sponge, we have found that miR-17 specifically blocks p21 expression, thereby promoting the expansion of the cortical neural progenitor pool. Our studies have revealed a mechanism of suppressing endogenous p21 expression by miR-17 in NPs.

Many studies have shown that p21 functions as a negative cell cycle regulator and induces cell cycle exit (13–15). In the nervous system, p21 has been shown to suppress self-renewal and proliferation of NSCs and NPs (16–19). An important question is how the p21 gene expression level is properly regulated *in vivo*. In the developing cortex, to produce sufficient numbers of NPs to form a functional brain, p21 expression has to be suppressed. Our study here has shown that miR-17, a member of the miR-17–92 cluster, is an important regulator of p21 expression and expansion of the cortical neural progenitor pool. miR-17 displays decreased expression in mouse cortices at embryonic and postnatal stages, which is opposite to that of p21, suggesting a reciprocal expression between miR-17 and its target p21. These results further suggest that higher expression of miR-17 in embryonic cortices is crucial for suppressing the p21 level and promoting NP proliferation, while low expression of miR-17 in postnatal cortices allows p21 expression to induce differentiation. Furthermore, we have found the direct silencing action of miR-17 on p21 *in vivo*, since co-expression of p21 and miR-17, but not miR-17 mutations, can block negative effects of p21 on cortical NP proliferation.

It is likely that silencing p21 by miR-17 in proliferative cells is a general rule. The targeting effects of miR-17 and p21 have been observed in oral carcinoma cells, acute myeloid leukemia cells, and Hodgkin’s lymphoma (39–41). Furthermore, miR-17 likely maintains the neural progenitor pool by regulating several targets. In this study, we have demonstrated miR-17 targeting effect on p21 expression. Here, we have not ruled out the likely possibility that the regulation of additional target genes by miR-17 contributes to its ability to promote NP proliferation. For instance, miR-17 has been shown to enhance NSC self-renewal by targeting Trp53inp1, a gene in the p53 pathway, to promote NP proliferation by silencing bone morphogenetic protein type II receptor (42, 43).

Therefore, proper self-renewal of NSCs and expansion of NPs in the developing cortex relies on balanced expression levels of cell cycle regulators such as p21. One of the mechanisms that control precise expression levels of these regulators is through the miRNA silencing regulation such as miR-17.

## Conflict of Interest Statement

The authors declare that the research was conducted in the absence of any commercial or financial relationships that could be construed as a potential conflict of interest.
